# Seasonal pattern of psychiatry service utilization in a tertiary care hospital

**DOI:** 10.4103/0019-5545.33254

**Published:** 2007

**Authors:** Gurvinder Pal Singh, B. S. Chavan, Priti Arun, Ajeet Sidana

**Affiliations:** Department of Psychiatry, Govt. Medical College and Hospital, Sector - 32, Chandigarh, India

**Keywords:** Mental and behavioral disorders due to psychoactive substance use, mood disorders, neurotic stress-related and somatoform disorders, schizophrenia, schizotypal and delusional disorders

## Abstract

**Background:**

Seasonal and monthly variations in utilization of psychiatric services have been inadequately studied in India.

**Aims:**

This study sought to determine the pattern of psychiatric services utilization by patients with four broad categories of diagnosis (mood disorders (F30-39): neurotic stress-related and somatoform disorders (F40-48), schizophrenia, schizotypal and delusional disorders (F20-29) and mental and behavioral disorders due to psychoactive substance use (F10-19) in different seasons and months of the last six years.

**Materials and Methods:**

We conducted a teaching hospital data-based study of new patients diagnosed with psychiatric illness in the department of psychiatry, Government Medical College and Hospital, Chandigarh. Four diagnostic groups consisting of 12058 psychiatric patients who had been diagnosed and treated in the department of psychiatry of this institute from 1999-2004 were included in this evaluation. Bed occupancy rate (BOR), average length of stay (ALOS) of inpatients and seasonal index were determined. Information about weather variables (mean daily temperature, mean rainfall) was collected from the meterological department of Chandigarh.

**Results:**

Psychiatric services were utilized by 31.1% of patients with mood disorders in the summer and by 34.23% of patients with neurotic, stress-related and somatoform disorders in the autumn. Statistical analysis revealed significant difference in new cases of these two groups of disorders in different seasons.

**Conclusion:**

Our study showed a significant relationship between utilization of psychiatric patients especially with mood disorders and neurotic, stress related and somatoform disorders with season (summer and autumn respectively).

Hospital utilization denotes the manner in which a certain community makes use of its hospital resources.[1] Psychiatric service utilization indices are relatively less known in our country. Treatment-seeking behaviour of a patient is sensitive to seasonal and environmental conditions, which are very variable in India. Seasonal and climate factors must be recognized for their potential effect on performance-monitoring systems focused on hospital utilization.[2] Indian researchers must consider broader contextual variables such as season and climate which influence the determinants of health care utilization and psychiatric hospitalization costs.

In an international study, effect of season on the length of hospital stay was studied among inpatients with psychiatric disorders.[3] The results revealed that mean monthly admission rates were significantly higher during the summers and patients' mean monthly admission rates correlated with mean maximal monthly environmental temperature. Morken *et al.*[4] examined monthly variations in admissions for mania and depressions. The authors found that male patients with depression had a significant monthly variation, the highest peak admission being in April. In contrast to these studies, Daniels *et al.*[5] reported that no significant seasonal variation was found in admissions with schizophrenia, depression or bipolar disorder in Tasmania. In a Brazilian study, a seasonal pattern in hospital admission was observed only for mania in women.[6] In a multi-ethnic international study, the Asian group was the only ethnic group that showed significant seasonal variation in depression.[7]

In India, Reddy *et al.*[8] studied the utilization of psychiatric service in teaching hospitals and found that the average length of stay of psychiatric patients was 20.9 days (range = 6-46 days). In another Indian study, the duration of stay was found to be less than two weeks.[9] In these studies, seasonal and monthly patterns of hospital data were not evaluated. No information about the effect of environmental factors on hospital utilization has been documented in studies from the Indian subcontinent. Previously, few efforts have been made to study the seasonal patterns of various service utilization indices from any part of India. Hence, the present study was planned with the following objectives:

- To study the seasonal and monthly variations in the number of new patients' visits with specified psychiatric diagnosis.
- To study the bed occupancy rate (BOR) and the average length of stay (ALOS) of these patients in different months of the year.

## MATERIALS AND METHODS

### Setting

This study was conducted in the department of psychiatry of the Govt. Medical College and Hospital, Chandigarh. This teaching hospital is a tertiary care health institution, which provides mental health services in a general healthcare setting. This hospital caters to the mental health needs of patients hailing from Chandigarh, Punjab, Haryana and Himachal Pradesh. In the department, patients are evaluated in detail first by a senior resident (Psychiatrist) in the psychiatry (outpatient department) OPD and later discussed with a consultant psychiatrist for the final diagnosis. Diagnosis of the patient is made according to the International Classification of Diseases (ICD-10, World Health Organization (WHO), 1992) criteria.[10] In this department, monthly chart meetings are held where a summary of all cases seen in a month is discussed. The summary includes the number of patients seen (both old and new), source of referral, sociodemographic variables and diagnosis. For the purpose of this study, additional variables were included in the monthly review of medical records.

### Sample of the study

All new patients of psychiatric disorder who reported to the department during last six years (January 1999 - December 2004) formed the study population for the present study. Four diagnostic groups, i.e., mood disorders (F30-39), neurotic stress-related and somatoform disorders (F40-48), schizophrenia, schizotypal and delusional disorders (F20-29) and mental and behavioral disorders due to psychoactive substance use (F10-19), have been included in the present study. Since the information about clinical variables was recorded on a semistructured performa and was discussed monthly in department chart meeting, the information was considered both adequate and reliable. The inclusion criteria for this study were:

1. Patients should fulfill the diagnostic criteria of ICD-10 (WHO, 1992).
2. Chart analysis and case record of the patient should be complete.
3. The patient should not have any other comorbid psychiatric, medical or neurological illness.

### Procedure

The details of all the new patients from January 1999 to December 2004 were collected on a semistructured proforma. Similar information was collected about all the patients admitted in the psychiatric ward during this time period. The year was divided into four seasons of equal lengths, namely, spring (February-April), summer (May-July), autumn (August-October) and winter (November-January). For the patients admitted in the psychiatric ward, the BOR and ALOS were determined monthly and annually. Weather information (mean daily maximum and minimum temperatures and mean rainfall) of different months was collected from the meterological department, Chandigarh to study any correlation of these variables with different psychiatric disorders.

### Analysis

The statistical methods used in this study were frequency distribution and chi-square test. Chi-square test was used to compare the seasonal numbers of new patients of different diagnostic groups. This was done to test whether there is a statistically significant difference in the observed distribution of new patients among the four seasons of the year. BOR, ALOS and the seasonal index was determined by the following formulae:

- $\mathrm{BOR}=\frac{\mathrm{Average daily census}\times 100}{\mathrm{Bed Complement}}$
- $\mathrm{ALOS}=\frac{\mathrm{Number of inpatients in day care}}{\mathrm{Total no. of discharges}}$
- $\mathrm{Seasonal index}=\frac{\mathrm{Quarterly average}\times 100}{\mathrm{General average}}$

Pearson's product moment correlation analysis was used to find the association between environmental temperature and the number of patients with different diagnoses.

## RESULTS

Between January 1999 and December 2004, 12,058 new patients diagnosed with four major psychiatric disorders (mood disorders; neurotic stress-related and somatoform disorders; schizophrenia, schizotypal and delusional disorders and mental and behavioral disorders due to psychoactive substance use) visited the department. The majority of these patients were suffering from neurotic stress-related and somatoform disorders (n = 4463, 37.01%) followed by 35.46% of patients with mood disorders (n = 4276). Patients with schizophrenia, schizotypal and delusional disorders constituted 11.72% (n = 1414) of the total study population. Patients diagnosed with mental and behavioral disorders due to psychoactive substance use constituted 15.79% (n = 1905) of the study population. The peak incidence of patients with mood disorders was observed from May to July. The maximum number of patients with neurotic stress-related and somatoform disorders came in July to September. BOR (%) was maximum in the months of June and July and ALOS was 15.41 days in the month of May.

Data was further analyzed comparing the recording of new cases in different seasons of the year. Among patients with mood disorders, summer was the season of the highest number of patient visits (31.1%) followed by the autumn (27.20%). The lowest number of new patients was recorded in the winter (19.9%). Among patients with neurotic, stress-related and somatoform disorders, autumn was the season of the highest number of patients (34.23%) followed by the summer. In comparison, there was no marked seasonal pattern in the visits by patients with schizophrenia, schizotypal and delusional disorders (F20-29) in this study (summer 25.3%; winter 24.5%; autumn 27.4%; spring 22.8%). Of the new cases of patients with mental and behavioral disorders due to psychoactive substance use (F10-19), 29.18% were recorded in the summer, 26.61% in the autumn and 23.14% in the spring.

Seasonal data was compared annually for the patients with these four different psychiatric disorders. Time series analysis of patients with mood disorders from 1999-2004 revealed that there was a statistically significant difference in the number of new cases in the four season of the year (chi-square value = 35.68, df = 15, *P* < 0.001) [Table 1]. Seasonal variation analysis of patients with neurotic stress-related and somatoform disorders also showed a similar statistically significant difference (Chi-square value = 84.26, df = 15, *P* < 0.001) [Table 2]. Annual analysis of patients with schizophrenia, schizotypal and delusional disorders did not show any statistically significant difference in the different seasons of the six years (Chi-square value = 18.52. df = 15, *P* = 0.236). Yearly seasonal variation analysis of patients with mental and behavioral disorders due to psychoactive substance use did not reveal any statistically significant difference (Chi-square value = 18.36, df = 15, *P* = 0.244). The daily mean temperature in Chandigarh was persistently highest in the month of June during the study period. Correlation analysis between the number of mood disorder patients and mean daily temperature (maximum) revealed a highly statistically significant value (*P* < 0.0001).

**Table 1:** Time series analysis of patients with mood disorders (F30-39) (n = 4276)

Year	Season			
	
	Winter (*n*)	Spring (*n*)	Summer (*n*)	Autumn (*n*)
1999	107	117	108	126
2000	94	130	143	122
2001	143	150	209	189
2002	163	143	245	212
2003	166	185	278	261
2004	183	238	348	252
Total	856	963	1331	1162
Average	142.66	160.50	221.83	193.66
Seasonal index	79.4	89.33	123.47	107.79
Chi-square	35.675	df = 15;		
value		*P*<0.001		

**Table 2:** Time series analysis of patients with neurotic stress-related and somatoform disorders (F40-48) (n = 4463)

Year	Season			
	
	Winter (n)	Spring (n)	Summer (n)	Autumn (n)
Seasonal index	126.28	109.39	95.46	80.24
1999	79	115	142	214
2000	121	107	159	218
2001	133	162	241	242
2002	159	188	204	194
2003	163	157	149	158
2004	122	168	133	197
Total	777	897	1028	1223
Average	126.28	109.39	95.46	80.24
Seasonal index	129.5	149.5	171.33	203.83
Chi-square	84.257	df = 15;		
value		*P*<0.001		

## DISCUSSION

The objective of this study was to determine the pattern of new psychiatric patients with different psychiatric disorders seeking treatment in various months and seasons of the year. This study was conducted in the department of psychiatry, Govt. Medical College and Hospital, Chandigarh. Chandigarh is located on the globe at 30 degrees, 44 inches latitude (north) and 76 degrees, 48 inches longitude (east) in the foot of the Shivalik range of the Himalayas. The department of psychiatry is located on the main highway of Chandigarh, hence, a large number of patients from different states have visited this department for mental health services in the last six years.

The main interpretation of our observation is that new patients predominately visited and received diagnosis of mood disorders in the summer season while neurotic stress-related and somatoform disorders were predominantly recorded in the autumn. These findings suggest that the number of patients belonging to a specific diagnostic group seeking treatment may be uniquely influenced by the season. The possible relationship between the seasons (summer, autumn) and the months (May-July; July-Sept.) between two major diagnostic groups (mood disorders and neurotic stress-related and somatoform disorders) is elucidated in the present study. Although the study has described the utilization of treatment facilities by mentally ill patients in terms of attendance at the OPD as well as hospital admission and length of stay, it can be hypothesized that a large number of these patients might have had illness of recent onset or may have relapsed recently due to seasonal variations.

Many international studies of an average duration of 5-6 years also reported the effects of season on hospital utilization statistics.[3,4,6,7] Similarly, our study has shown persistent findings for six consecutive years. ALOS and BOR were high in our study in the summer months. Seasonal variations have been observed in emergency psychiatric visits of the patients and a greater number of visits have been documented in the summer.[11] Kecskes *et al.*[12] reported that ALOS of inpatients had a significant relationship with the season. In the present study, the peak incidence of visits by patients with mood disorders was observed from May to July. In Chandigarh, the weather conditions show many variations during these months. The heat wave, which in fact starts in the first part of May, continues to sweep Chandigarh and other parts of north India with the mercury hovering well above the 40°C mark. The information received from the meteriological department revealed that the intensity of heat is maximum in the month of June when the sun is in its annual northward motion and reaches the Tropic of Cancer (23.5 degrees north latitude) and in mid-June, the sun turns about to move southward.

Our study showed that the number of patients with mood disorders has been consistently found to be maximum in the month of June during which the mean daily temperature is also high. Correlation analysis revealed a highly statistically significant relationship between the number of mood disorders and the maximum daily temperature. The build-up of the heat-wave peaks for a month or more from the first week of June onwards all over North India. In the summer, particularly from May to June, there is no rainfall in northern India and hot winds known as “loo, blow in a westerly direction. When humid air comes in contact with the “loo, it causes storms known as Norwesters. All these factors together contribute to the building up of above-normal temperatures. The prevailing unusual heat wave in North India is a localized phenomenon, but it could also be part of a much larger climate-changing regime. BOR of the admitted patients was also high in these months.

No statistically significant difference was found in the present study in the seasonal distribution of patients with schizophrenia, schizotypal and delusional disorders and mental and behavioral disorders due to psychoactive substance use during the last six years. In this study, four broad diagnostic groups were included. Our study is unique in the way that it has attempted to investigate the seasonal influence on service utilization by patients with these different types of psychiatric disorders. No national or international study has studied the seasonality pattern in patients' visits with neurotic stress-related and somatoform disorders. Even though this study has a few limitations (being hospital data-based, without gender and clinical subtypes or variables), its findings cannot be ignored. The unmet needs of this field of healthcare call for greater interest in this area. Importance should be given to a psychiatric patient's report of being affected by the seasonal, monthly and weather variables. We would suggest the replication of our study using more clinical details of patients with different psychiatric disorders.

The observations of the present study have a number of implications. Firstly, the findings may justify the need for more manpower in psychiatric wards during those months when the numbers of patients are at their peak. Staff can plan to go on leave so as to ensure proper availability of adequate staff during these months. Also, the administrator of the hospital can be sensitized to make more beds available for the care of mentally ill persons during the peak months. Secondly, for calculating the annual requirement of medication for the patients, the average requirement should be considered for the whole year and not for a particular month. Thirdly, this study's findings may help in planning research and to calculate the intake period for patients with mood disorders, neurotic stress-related and somatoform disorders.

## CONCLUSION

Our study showed a statistically significant relationship between seasonal visits in the summer and autumn and the number of patients with mood disorders, neurotic stress-related and somatoform disorders patients being recorded who utilize psychiatric services in a tertiary psychiatric institution. The peak incidence of mood disorders was found in the months of June and July during the last six years. High numbers of patients with neurotic stress-related and somatoform disorders availed of the psychiatric service in the months of August and September. In contrast, although some seasonal and monthly variations were evident among patients wih schizophrenia, schizotypal and delusional disorders and mental and behavioral disorders due to psychoactive substance use, they did not reach a statistically significant value. In the psychiatric ward, the BOR was highest in the summer and the ALOS was high in the summer and autumn months. The present study may indicate that persistently high environmental temperatures may be a contributing factor in this relationship between summer and autumn with the visit of patients with mood disorders. Further research is needed to determine the effect of different weather variables in different seasons with specific subtypes of mood disorders and neurotic stress-related and somatoform disorders.
